# Corticosteroid-Sparing Effect of Chromoglycate Sodium and Nedocromil

**DOI:** 10.1155/S0962935194000712

**Published:** 1994

**Authors:** A. F. Capristo, M. Miraglia del Giudice Jr, C. Alfaro, N. Maiello

**Affiliations:** 1 Paediatric Clinic University of Naples via Sant'Andrea delle Dame Naples 4-1-80100 Italy

## Abstract

The most appropiate management for bronchial asthma is the control of airway inflammation. Corticosteroids are the most effective anti-inflammatory drugs available, but they have a number of side effects; most of these are dose-dependent. In children, asthma control should be accomplished with low steroid doses possibly given by inhalation. In a double-bind placebo-controlled crossover study a group of children with mild to moderate asthma received NED 16 mg/day or BDP 400 μg/day. Values for FEV_1_, PEF, symptoms use ofbronchodilators overlapped, whereas bronchial hyper-responsiveness assessed by histamine bronchoprovocation challenge was better with BDP than NED. In another case, one boy with high bronchial hyper-reactivity assessed by provocation test with hypertonic solution, experienced a significant improvement only after 2 weeks of therapy with Deflazacort (2 mg/Kg/day) followed by 4 months on combined treatment with NED (16 mg/day) and BDP (300 μ/day). Authors conclude that NED could have a steroidsparing effect over long-term use.


Mediators of Inflammation 3, S25–S30 (1994)

MOIA7A

### Corticosteroid-sparing effect of chromoglycate sodium and nedocromil

**A. F. Capristo, M. Miraglia del Giudice Jr,[CA] C. Alfaro and N. Maiello**

Paediatric Clinic, University of Naples, via Sant'Andrea delle Dame, 4-1-80100 Naples, Italy

[CA] Corresponding Author

**The most appropiate management for bronchial asthma is the control of airway inflammation. Corticosteroids are the most effective anti-inflammatory drugs available, but they have a number of side effects; most of these are dose-dependent. In children, asthma control should be accomplished with low steroid doses possibly given by inhalation. In a double-bind placebo-controlled cross-over study a group of children with mild to moderate asthma received NED 16 mg/day or BDP 400 μg/day. Values for $FEV_1$, PEF, symptoms use of bronchodilators over-lapped, whereas bronchial hyper-responsiveness assessed by histamine bronchoprovocation challenge was better with BDP than NED. In another case, one boy with high bronchial hyper-reactivity assessed by provocation test with hypertonic solution, experienced a significant improvement only after 2 weeks of therapy with Deflazacort (2 mg/Kg/day) followed by 4 months on combined treatment with NED (16 mg/day) and BDP (300 μg/day). Authors conclude that NED could have a steroid-sparing effect over long-term use.**

**Key words:** NED, Asthma, Bronchial Hyper-reactivity, Inhaled Corticosteroids.

It is now clear that the most appropriate management of bronchial asthma is to control airway inflammation.[1,2] The drugs able to reduce inflammation in asthmatic children are in the following order of decreasing activity: systemic steroids; inhaled steroids (IS); nedrocromil sodium (NED); sodium cromoglycate (SGC); and probably ketotifen.[3–5]

The anti-inflammatory role of the new long-acting beta-2 agonist, *salmeterol*, has not been established because a 12-week treatment period of doses effective in controlling symptoms did not show any significant effect on mast cells, eosinophils, T-cells or the epithelium of bronchial mucosa and submucosa.[6]

### Anti-inflammatory drugs and mild-to-moderate asthma

The international guidelines about asthma management suggest regular anti-inflammatory therapy should be given in mild persistent, moderate and severe asthma. Only mild episodic asthma should be treated exclusively with environmental control and beta-2 agonist on demand. However, it is not certain which anti-inflammatory agent should be used as first-line therapy.[7–9]

Corticosteroids are the most effective anti-inflammatory drugs,[9] but they have a number of side effects (Tables 1 and 2). Inhaled steroid therapy is effective in controlling asthma without the side effects associated with systemic administration, although recent reports have shown that the dose of 400 mcg/day is not 'safe', since growth may be affected,[10] the hypophysio-pituitary-adrenal axis may be suppressed[11,12] and bone metabolism may be influenced.[13]

**Table 1.** Side effects of inhaled corticosteroids

| Local side effects |
|---|
| • Cough |
| • Bronchostenosis |
| • Oral candidosis |
| • Atrophic glossitis |
| • Haemorrhagic angina |
| • Dysphonia |
| • Chronic oesophagitis |
| • Laryngeal candidosis |

**Table 2.** Side effects of inhaled corticosteroids

| Systemic side effects |
|---|
| • HPA suppression |
| • Growth delay in asthmatic children |
| • Interference in bone formation |
| • Reduction of circulating eosinophils and lymphocytes |
| • Metabolic effects |
| • Cataract |
| • Cushing syndrome faeces |
| • Derma thinning |
| • Purpura |

Sodium cromoglycate (SCG) may be the drug of choice in children with mild-to-moderate asthma, because of its optimal ratio between effectiveness and safety.[14] SCG is able to control both clinical symptoms and bronchial hyper-responsiveness; it is also active in reducing the number of inflammatory cells in the asthmatic airway.[15–18] SGC is equally effective as low doses of inhaled steroids, but has the advantage of being safer.[19–21]

© 1994 Rapid Communications of Oxford Ltd

**Table 3.** Clinical features of children recruited for the trial in treatment group 1*

| | |
|---|---|
| Age, mean (range) | 10.46 (8–13 years) |
| Sex | 7 F and 8 M |
| FEV1 %, mean (range) | 73.39 (70.2–79.1) |
| % Reversibility after Beta-2, mean (range) | 19.76 (16.7–22.5) |
| PC20 Histamine, mean (range) | 670 (250–1200) |
| Allergic asthma | 15/15 (Dermat. +++) |

*Trial started with BDP 400 mcg/day + placebo for 6 weeks, then cross-over with nedocromil sodium 16 mg (NED) + placebo.

**Table 4.** Clinical features of children recruited for the trial in treatment group 2*

| | |
|---|---|
| Age, mean (range) | 10.6 (8–13 years) |
| Sex | 5 F and 10 M |
| FEV1 % mean (range) | 70.02 (69.3–78.5) |
| % Reversibility after Beta-2, mean (range) | 19.56 (16.7–22.7) |
| PC20 Histamine, mean (range) | 653 (400–1000) |
| Allergic asthma | 13/15 (Dermat. +++) |

*Trial started with NED 16 mg/day + placebo for 6 weeks, then cross-over with BDP 400 mcg/day + placebo.

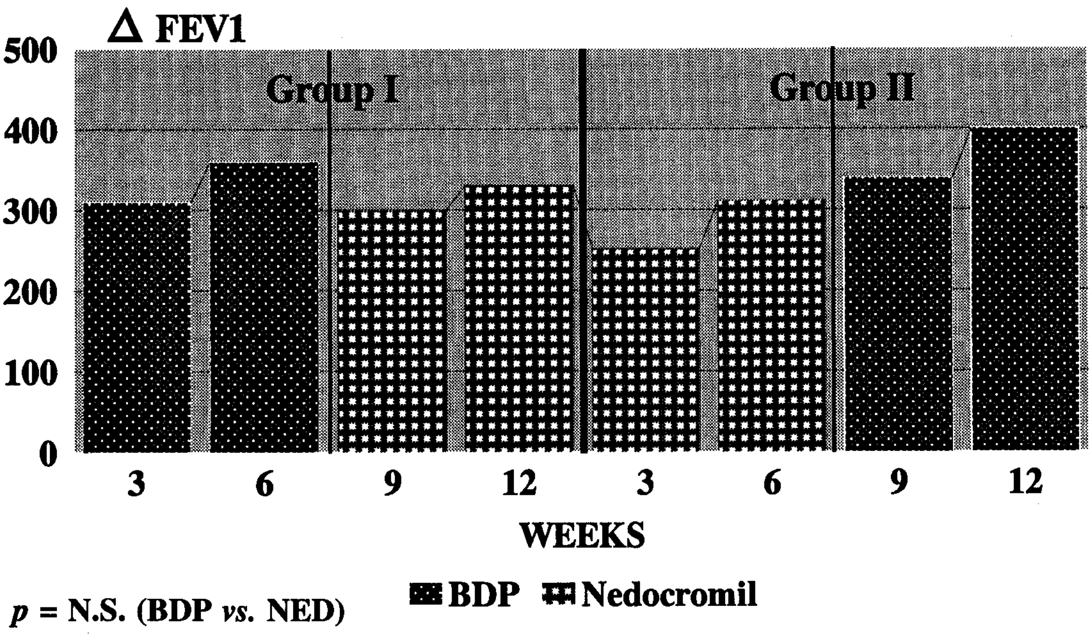

FIG. 1. BDP *vs.* nedocromil. Δ FEV1 (mean change from baseline) in the I and II groups treated.

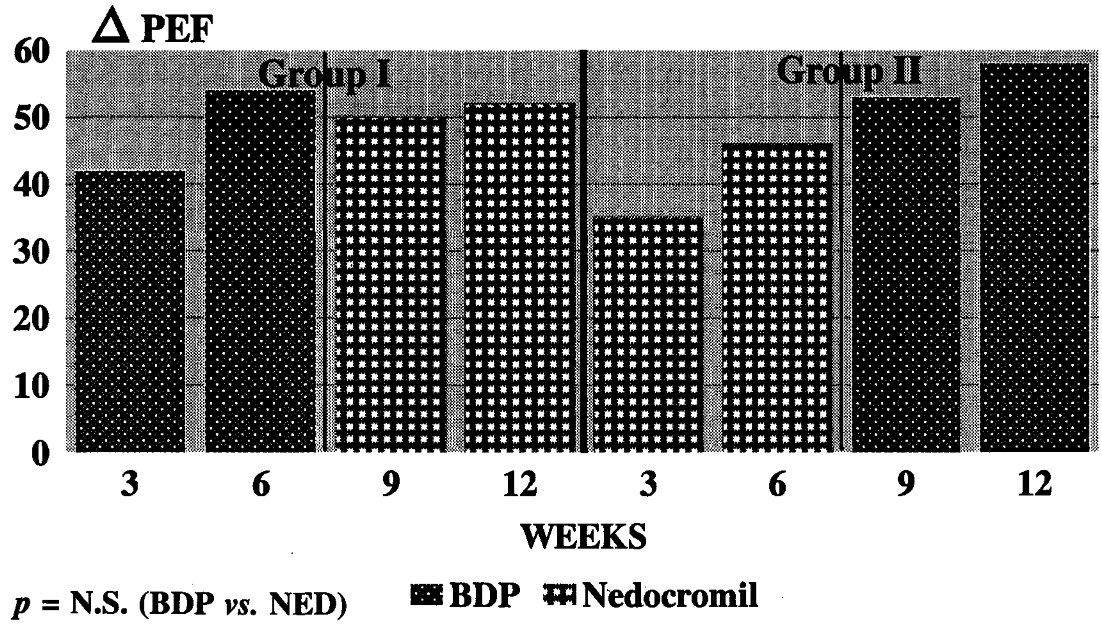

FIG. 2. BDP *vs.* nedocromil. Δ PEF (mean change from baseline) in the I and II groups treated.

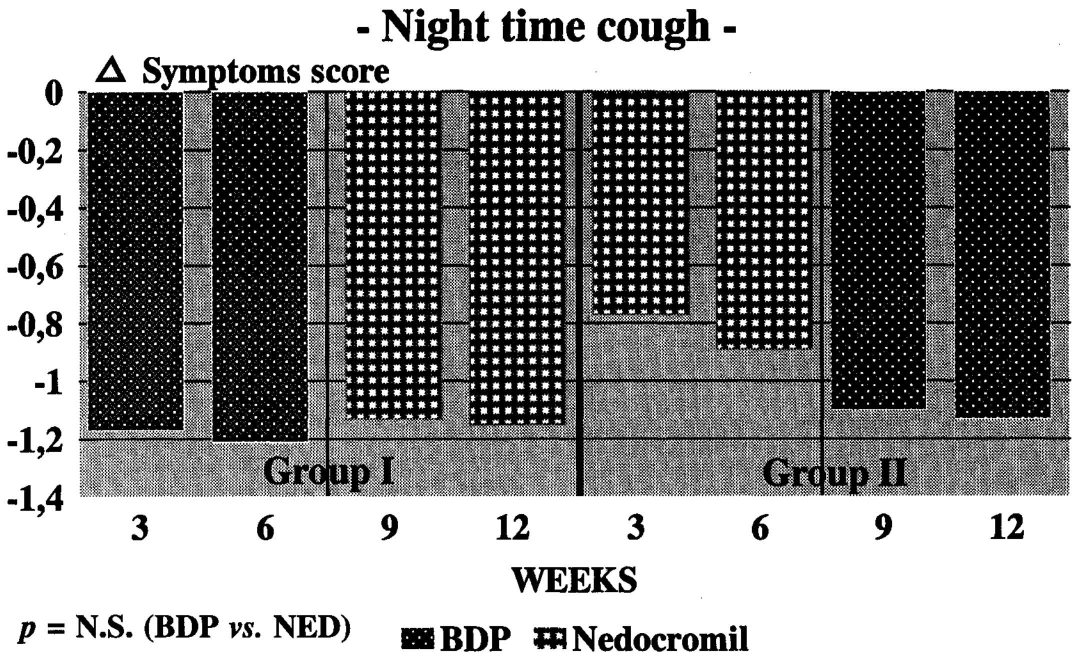

FIG. 3. BDP *vs.* nedocromil. Δ Symptoms score (mean change from baseline) in the I and II groups treated.

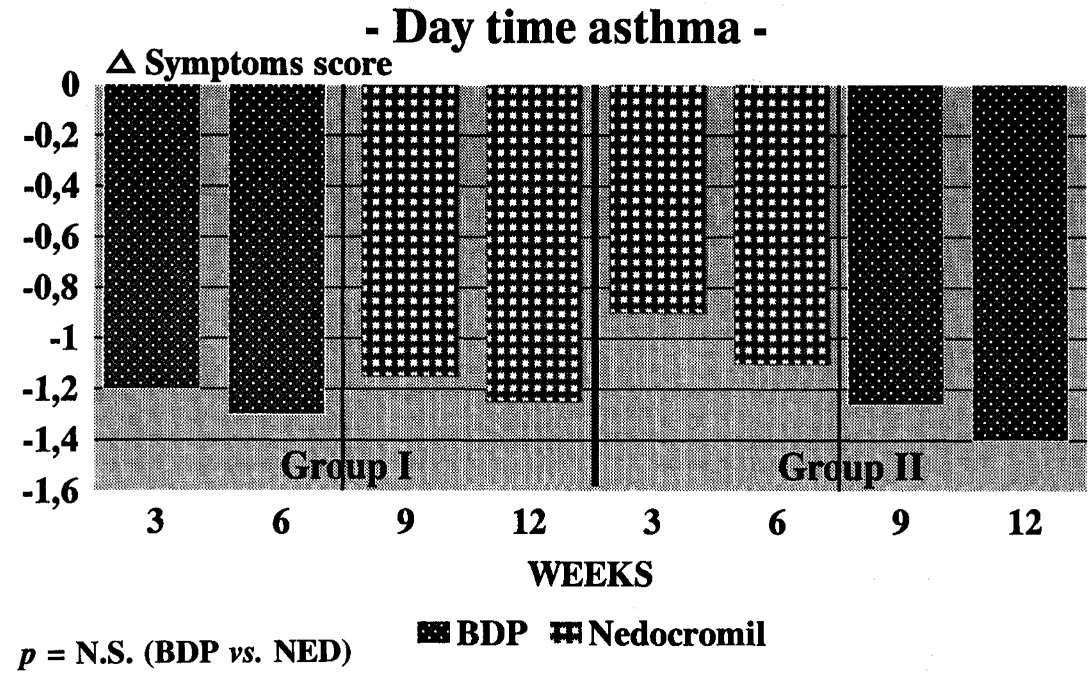

FIG. 4. BDP *vs.* nedocromil. Δ Symptoms score (mean change from baseline) in the I and II groups treated.

**Table 5.** Nedocromil sodium and steroids: reduction of corticosteroids therapy in adult asthmatic patients

| Authors (ref) | Treatment duration (weeks) | Nedocromil (dose, mg) | Steroid-sparing effect |
|---|---|---|---|
| Bone *et al.* (25) | 4 | 16 | yes |
| Svendsen and Jorgensen (26) | 8 | 16 | no |
| Orefice *et al.* (27) | 12 | 16 | yes |
| Paananen *et al.* (28) | 12 | 16 | no |
| Ruffin *et al.* (29) | 4 | 16 | yes |
| Goldin and Bateman (30) | 20 | 16 | no |
| Wong *et al.* (31) | 16 | 16 | yes |
| Boulet *et al.* (32) | 12 | 16 | no |

Nedocromil sodium (NED) has been shown to be equally or more effective than SCG as an anti-inflammatory agent, especially in intrinsic asthma.[22–24] In this paper, we report the results of a double-blind cross-over study comparing the effects of NED (16 mg/day) and BDP (400 mcg/day) on symptoms and bronchial hyper-responsiveness in 30 children with moderate asthma (Tables 3 and 4, and Figs 1–6). The patients had a wash-out off-therapy period for a minimum of 2 months. After a 2-week run-in period, children were randomly allocated to two similar treatment groups (Tables 3 and 4), receiving BDP (400 mcg/day) + placebo or NED (16 mg/day) +

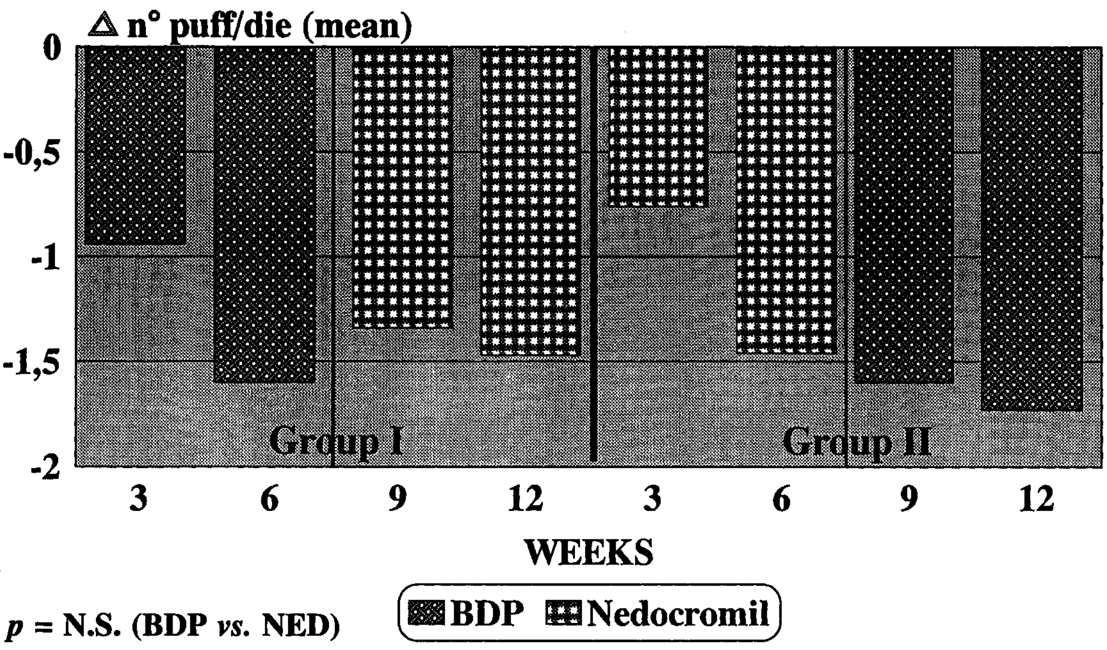

FIG. 5. BDP *vs.* nedocromil. Δ n° puff/die of Beta2 (mean change from baseline) in the I and II groups treated.

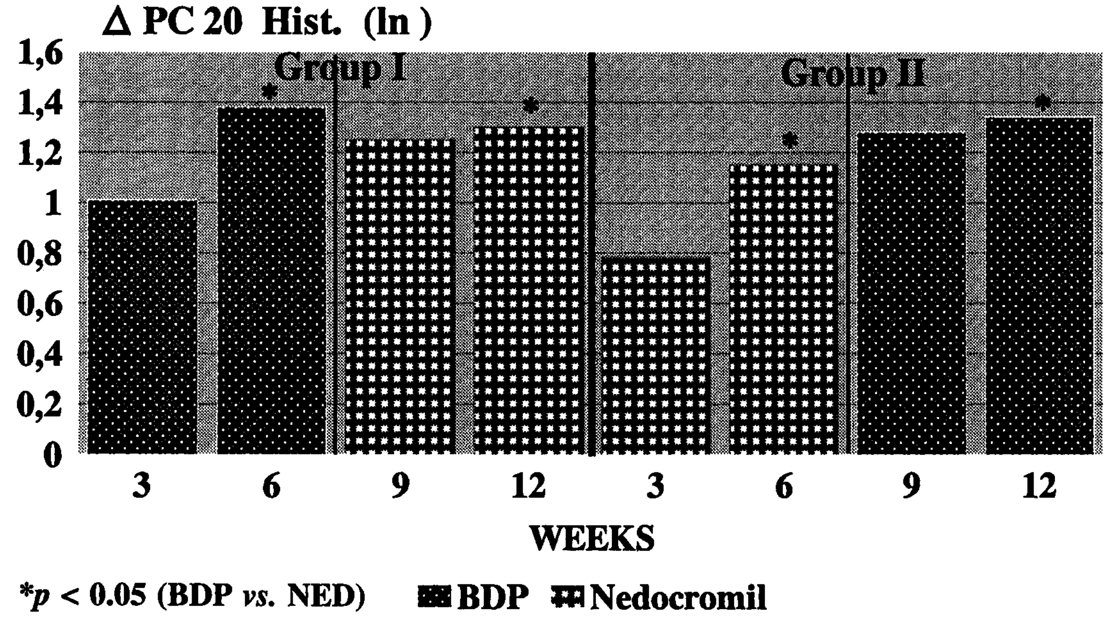

FIG. 6. BDP *vs.* nedocromil. Δ PC 20 Histamine (mean change from baseline) in the I and II groups treated.

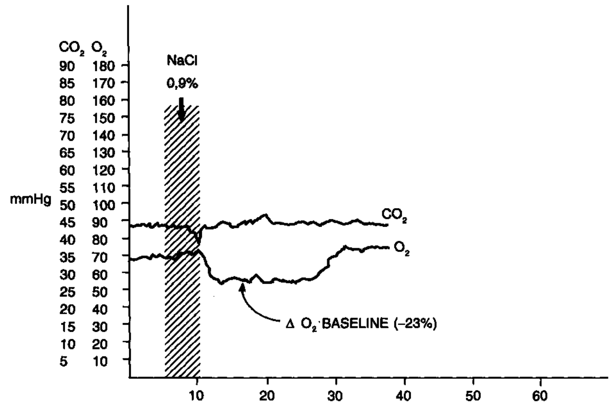

FIG. 7. Hyperosmolar solution challenge at baseline.

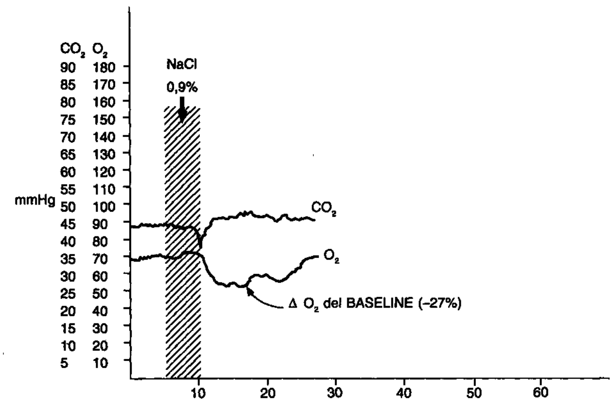

FIG. 8. Hyperosmolar solution challenge after 2 months' therapy with NED.

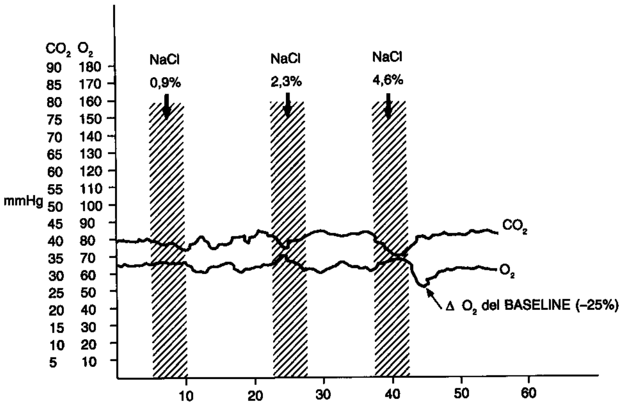

FIG. 9. Hyperosmolar solution challenge after 2 weeks' therapy with Deflazacort.

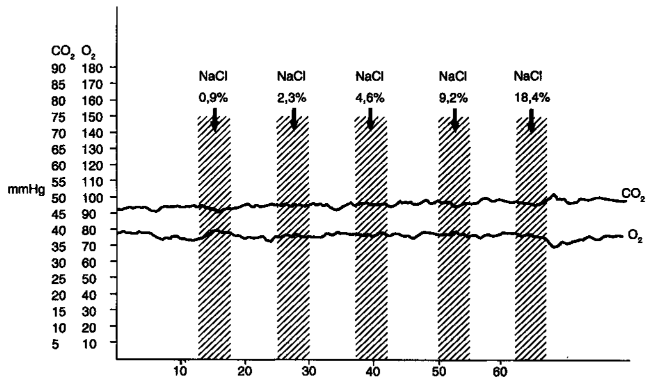

FIG. 10. Hyperosmolar solution challenge after 4 months' theapy with NED + BDP.

placebo, respectively, for 6 weeks in a cross-over design. Assessment was made by diary cards, bronchodilator use and intermittent clinical evaluation, spirometry and histamine bronchoprovocation challenge (at baseline and after 3, 6, 9 and 12 weeks). No significant difference (*t*-test analysis) was found between the two treatment groups (Figs 1–5), except that the PC20 histamine was significantly higher after 6 weeks in the corticosteroid group ($p > 0.05$) (Fig. 6).

Our study also showed that NED is not effective before 4 weeks of treatment. Furthermore, in an unpublished follow-up study, we found that the highest values of FEV1 occurred after 3 months of continuous treatment with NED.

In moderate to severe asthma, anti-inflammatory therapy with SCG or NED should be reinforced by increasing doses (i.e. SCG 40 mg × 4 times/day) and/or by increasing the times of administration[23] and/or by adding inhaled steroids. Dose adjustments of inhaled steroids should be made on the basis of clinical response, but high doses are not usually required because of the steroid sparing effect of SCG and NED.

It has been demonstrated that NED (16 mg/day) could replace BDP (330 mcg/day).[25] When overall control of asthma is achieved, inhaled steroids should be gradually reduced until the lowest effective dose is found or until their gradual but complete withdrawal.

Figures 7–10 show the results of provocation tests with hypertonic solution by monitoring $po_2$ and $pco_2$ with Gasthmatic, in an 8-year-old boy with moderate allergic asthma. The hyperosmolar solution challenge was performed with increasing concentrations of NaCl solution (2.3–4.6–9.2–18.4%) administered by ultrasonic nebuliser. At baseline, the patient already expressed significant derangements in $PO_2$ and $PCO_2$ on inhalation of normosmolar solution (Fig. 7). Two months after NED (16 mg/day), the findings were unchanged (Fig. 8). Fourteen days on Deflazacort (2 mg/Kg/day) brought about a reduction in bronchial inflammation (Fig. 9). The challenge became negative after four months of treatment with NED (16 mg/day)+BDP (300 mg/day) (Fig.10).

### Anti-inflammatory Drugs and Severe Asthma

In severe asthma, which is fortunately rare in childhood, corticosteroids are widely accepted as a first-line therapy.[9] When control of asthma appears to require continuous treatment with steroids, an alternative diagnosis should be first suspected, since a number of non-asthmatic conditions may cause wheezing or dyspnea (i.e. cystic fibrosis, congenital heart disease, recurrent aspiration).

When the diagnosis of asthma is certain, all the precipitating trigger factors (such as food allergens, cigarette smoke, occupational and environmental exposure, chronic sinusitis) should be identified and removed where possible. Before starting with steroid therapy, it is also necessary to control the adequacy of the previous therapeutic regimen, ensure the patient's compliance, and to control the inhalation technique and the aerosol delivery system output.

In cases requiring the use of steroids, inhaled steroids are the first choice treatment.[9] However, the therapeutic effects of inhaled steroids are dose-dependent, high-dose inhaled steroid therapy shows an increased risk of side effects, including adrenal and growth suppression.[11,12]

The minority of asthmatic children continuing to have an inadequate response to inhaled steroids need to go on to have oral administration. Oral steroids should be used at the lowest effective dose possible and be given on alternate days.[9] The ability of SCG and NED in reducing steroid requirements in steroid-dependent asthmatic patients is controversial, as shown in Table 5.[25–33] Dissimilar results in different trials could depend on the early suspension of sterinhaled steroids after their withdrawal, which lasts for about 3 months.[34] In asthmatic children who are dependent on oral steroids, drugs such as troleandomicin,[35] methotrexate,[36] cyclosporin,[37] and high dose i.v. immunoglobulins,[38] could be useful for their steroid-sparing effect. However, as there is not enough good and safe evidence in support of these drugs in childhood asthma, their use cannot yet be recommended.

### Conclusions

In most cases, bronchial asthma requires anti-inflammatory treatment which should be started as early as possible. Laitinen *et al.*[7] demonstrated a significant increase in eosinophils ($p < 0.001$), lymphocytes ($p < 0.001$) and plasma cells ($p < 0.001$) in bronchial mucosa, even in the mildest of asthmatic patients with a diagnosis made between 2 and 12 months ago. Since asthma is a chronic disease, anti-inflammatory therapy could be used for many years. Thus, SCG and NED should be considered as a first-line therapy because of their optimal safety profile. Corticosteroids should be reserved for patients whose symptoms are not completely controlled by courses of at least 2–3 months with SCG or NED used at dosages even higher than those conventionally suggested. Inhaled steroids, when they are required to control the disease, should be associated with SCG or NED in order to reduce the steroid dose.

### References

1. Holgate ST. Asthma: past, present and future. *Eur Respir J* 1993; **6**: 1507–1520.
2. National Heart, Lung and Blood Institute *International Consensus Report on Diagnosis and Management of Asthma* US Department of Health and Human Service, Publication No. 92-3091, June 1992.
3. Djukanovic R, Wilson JW, Britten KM, *et al.* Effect of an inhaled corticosteroid on airway inflammation and symptoms in asthma. *Am Rev Respir Dis* 1992; **145**: 669–674.
4. Trigg C, Manolitsas N, McAuley A, *et al.* A pilot comparative study of the effects of inhaled nedocromil sodium and albuterol on bronchial biopsies in asthma. *Am Rev Respir Dis* 1993; **147**: A522.
5. Diaz P, Galleguillos FR, Gonzales CM, *et al.* Bronchoalveolar lavage in asthma: the effect on sodium cromoglycate (cromolyn) on leucocyte counts, immunoglobulins and complement. *J Allergy Clin Immunol* 1984; **74**: 41–48.
6. Roberts JA, Bradding P. The effect of salmeterol therapy on mucosal inflammation in asthma. *Am Rev Respir Dis* 1992; **145**: A418 (abstract).
7. Laitinen LA, Laitinen A, Haahtela T. Airway mucosal inflammation even in patients with newly diagnosed asthma. *Am Rev Respir Dis* 1993; **147**: 697–704.
8. Szefler SI. Potential adverse effects of topical steroids. *Ann Meeting Am Coll Allergy Immunol* Chicago Illinois, 15 November 1992; 117–125.
9. Doull IJM, Freezer NJ, Holgate ST. Growth of asthmatic children on inhaled corticosteroids. *Am Rev Resp Dis* 1993; **147**: A265.
10. Padfiekd PL, Teelucksing S. Inhaled corticosteroids—the endocrinologist's view. *Eur Respir Rev* 1993; **3**: 449–500.
11. Phillip M, Aviram M, Leberman F, *et al.* Integrated plasma cortisol concentration in children with asthma receiving long term inhaled corticosteroids. *Ped Pulmonol* 1992; **12**: 84–89.
12. Sorva R, Turpeinem M, Juntunen-Backman K, *et al.* Effects of inhaled budesonide on serum markers of bone metabolism in children with asthma. *J Allergy Clin Immunol* 1992; **90**: 808–815.
13. Dukes MNG, Holgate ST, Pauwels RA. Report of an international workshop on risk and safety of asthma therapy. *Clin Allergy* 1994; **24**: 1–6.
14. Watanabe H. The effect of disodium cromoglycate against bronchial hyper-responsiveness in asthmatic children. *J Asthma* 1992; **29**: 117–120.
15. Shapiro GG, Furukawa CT, Pierson WE, *et al.* Double-blind evaluation of nebulized cromolyn, terbutaline and combination for childhood asthma. *J Allergy Clin Immunol* 1988; **81**: 449–454.
16. Cockcroft DW, Murdock KV. Comparative effects of inhaled salbutamol, sodium cromoglycate and beclomethasone dipropionate on allergen - induced early asthmatic response, late asthmatic response and increased bronchial responsiveness histamine. *J Allergy Clin Immunol* 1987; **79**: 734–738.
17. Shapiro GG, Sharpe M, De Rouen TA, *et al.* Cromolyn versus triamcinolone acetonide for youngest with moderate asthma. *J Allergy Clin Immunol* 1991; **88**: 742–748.
18. Kuzemko JA, Bedford S, Wilson L, *et al.* A comparison of beclo-methasone valerate aerosol and sodium cromoglycate in children with reversible airways obstruction. *Postgrad Med J* 1974; **50** (Suppl 4): 53–58.
19. Francis RS, McEnery G. Disodium cromoglycate compared with beclomethasone dipropionate in juvenile asthma. *Clin Allergy* 1984; **14**: 537–540.
20. Mitchel I, Paterson IC, Cameron SJ, *et al.* Treatment of childhood asthma with sodium cromoglycate and beclomethasone dipropionate aerosol singly or in combination. *Br Med J* 1976; **2**: 457–458.
21. Price JF. Corticosteroids and other anti-inflammatory agents in the treatment of children. *Eur Respir Rev* 1994; **4**: 27–32.
22. Bel EH, Timmers MC, Hermans J, *et al.* The long-term effects of nedocromil sodium and beclomethasone dipropionate on bronchial responsiveness to methacoline in non atopic asthmatic subjects. *Am Rev Respir Dis* 1990; **141**: 21–28.
23. de Jong JW, Postma DS, de Monchy JGR, *et al.* A review of nedocromil sodium in asthma therapy. *Eur Respir Rev* 1993; **3**: 511–519.
24. Bergman K, Bauer CP, Overlack A. A placebo controlled, blind comparison of nedocromil sodium and beclomethasone dipropionate in bronchial asthma. *Curr Med Res Opin* 1989; **11**: 533–542.
25. Bone MF, Kubik MM, Keaney P, *et al.* Nedocromil sodium in adults dependent on inhaled corticosteroids: a double blind, placebo controlled study. *Thorax* 1989; **44**: 654–659.
26. Svendsen UG, Jorgensen H. Inhaled nedocromil sodium as additional treatment to high dose inhaled corticosteroids in the management of bronchial asthma. *Eur Respir J* 1991; **4**: 992–999.
27. Orefice U, Struzzo P, Dorigo P, *et al.* Long term treatment with sodium cromoglycate, nedocromil sodium and beclomethasone dipropionate reduces bronchial hyper-responsiveness in asthmatic subjects. *Respiration* 1992; **59**: 97–101.
28. Paananen M, Karakorpi T, Kreus KE. Withdrawal of inhaled corticosteroid under cover of nedocromil sodium. *Eur J Respir Dis* 1986; **69** (Suppl 147): 330–335.
29. Ruffin R, Alpers JH, Kroemer DK, *et al.* A 4 week Australian multicentre study of nedocromil sodium on asthmatic patients. *Eur J Respir Dis* 1986; **69** (Suppl 147): 336–339.
30. Goldin JG, Bateman ED. Does nedocromil sodium have a steroid-sparing effect in adult asthmatic patients requiring maintenance oral corticosteroids? *Thorax* 1988; **43**: 982–986.
31. Wong CS, Cooper S, Britton GR, *et al.* Steroid-sparing effect of nedocromil sodium in asthmatic patients taking high doses of inhaled steroids. *Thorax* 1991; **46**: 768p–769p.
32. Boulet LP, Cartier A, Cockcroft DW, *et al.* Tolerance to reduction of oral steroid dosage in severely asthmatic patients receiving nedocromil sodium. *Respir Med* 1990; **84**: 317–323.
33. Juniper EF, Kline PA, Vanzieleghem MA, *et al.* Reduction of budesonide after a year of increased use: a randomized controlled trial to evaluate whether improvements in airway responsiveness and clinical asthma are maintained. *J Allergy Clin Immunol* 1991; **87**: 483–489.
34. Toogood JH, Jennings B, Lefcoe NM. A clinical trial of combined cromolyn/beclomethasone treatment for chronic asthma. *J Allergy Clin Immunol* 1981; **67**: 317–324.
35. Wald JA, Friedman BF, Farr RS. An improved protocol for the use of troleandomycin (TAO) in the treatment of steroid-requiring asthma. *J Allergy Clin Immunol* 1986; **78**: 36–43.
36. Shiner RJ, Nunn AJ, Chung KF, *et al.* Randomised, double-blind, placebo controlled trial of methotrexate in steroid-dependent asthma. *Lancet* 1990; **336**: 137–140.
37. Szczeklik E, Nizankowska E, Dworski B, *et al.* Cyclosporin for steroid-dependent asthma. *Allergy* 1991; **46**: 312–315.
38. Mazer BD, Gelfand EW. An open-label study of high-dose intravenous immunoglobulin in severe childhood asthma. *J Allergy Clin Immunol* 1991; **87**: 976–983.
